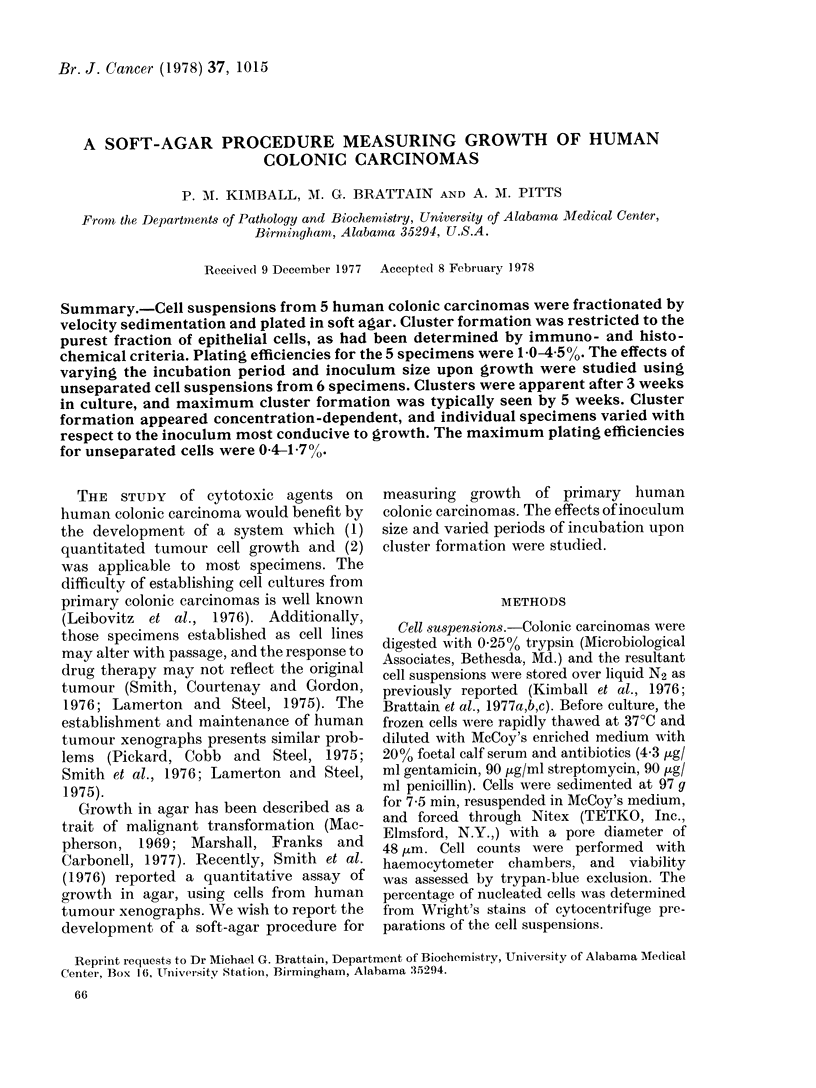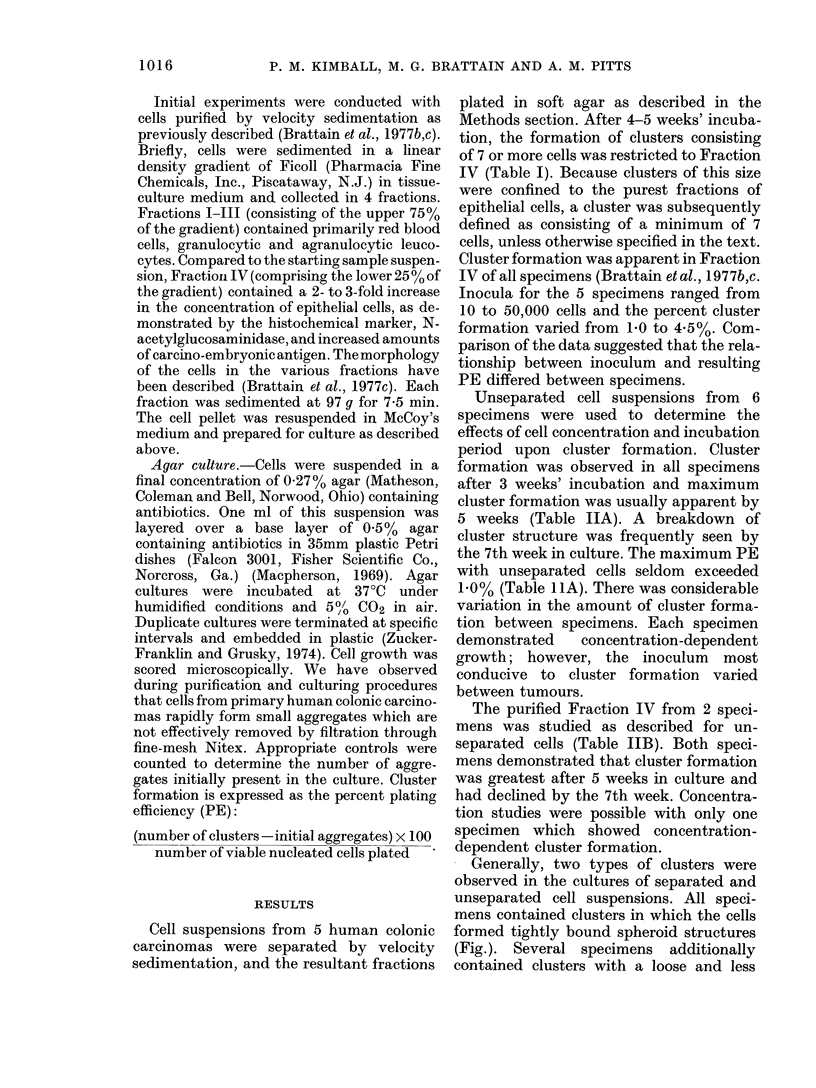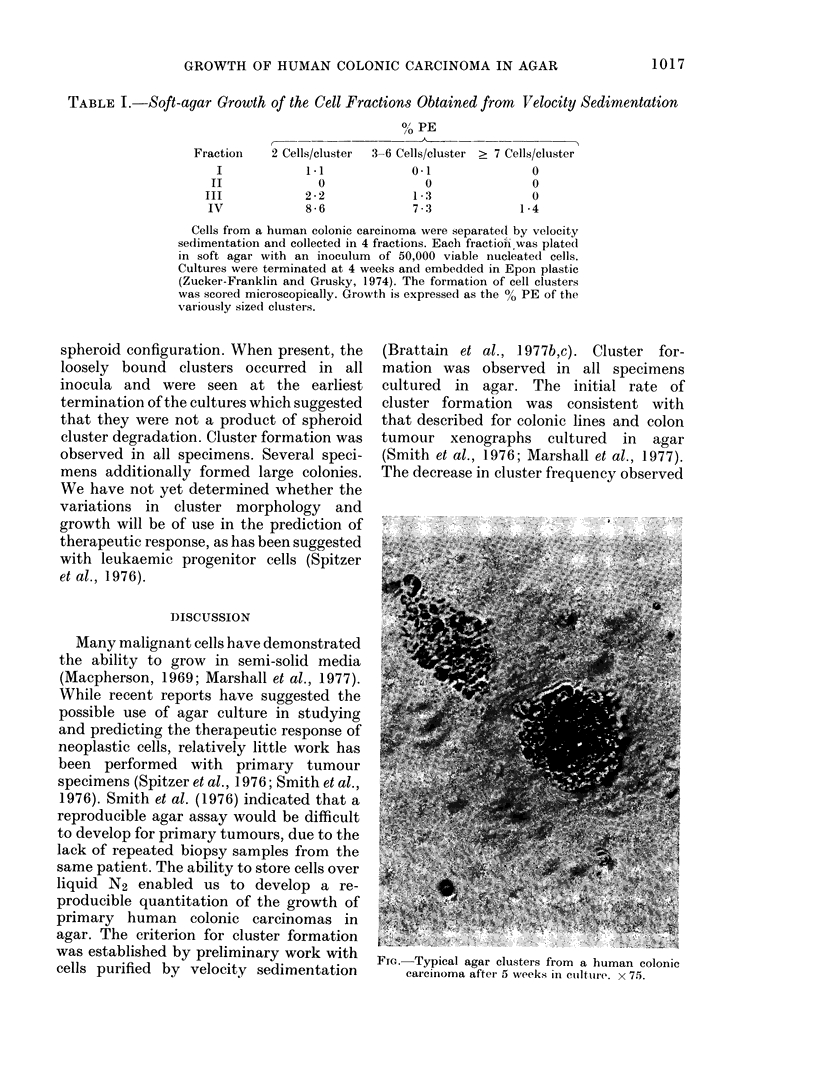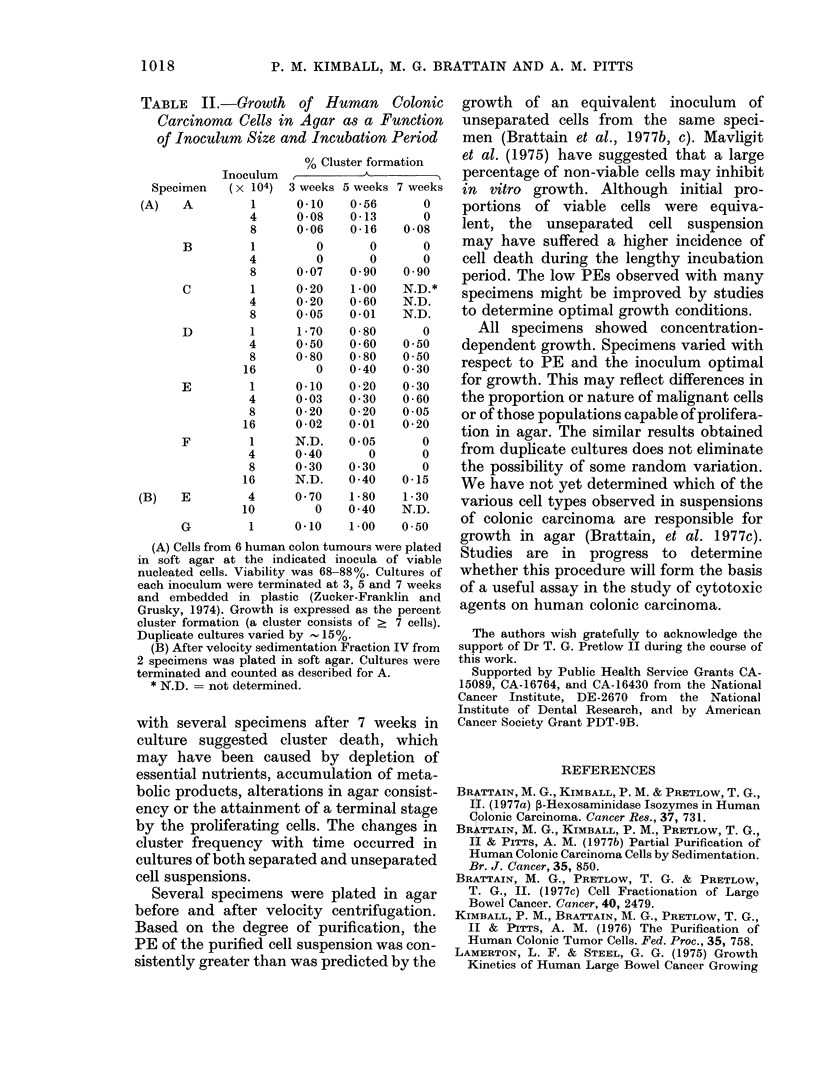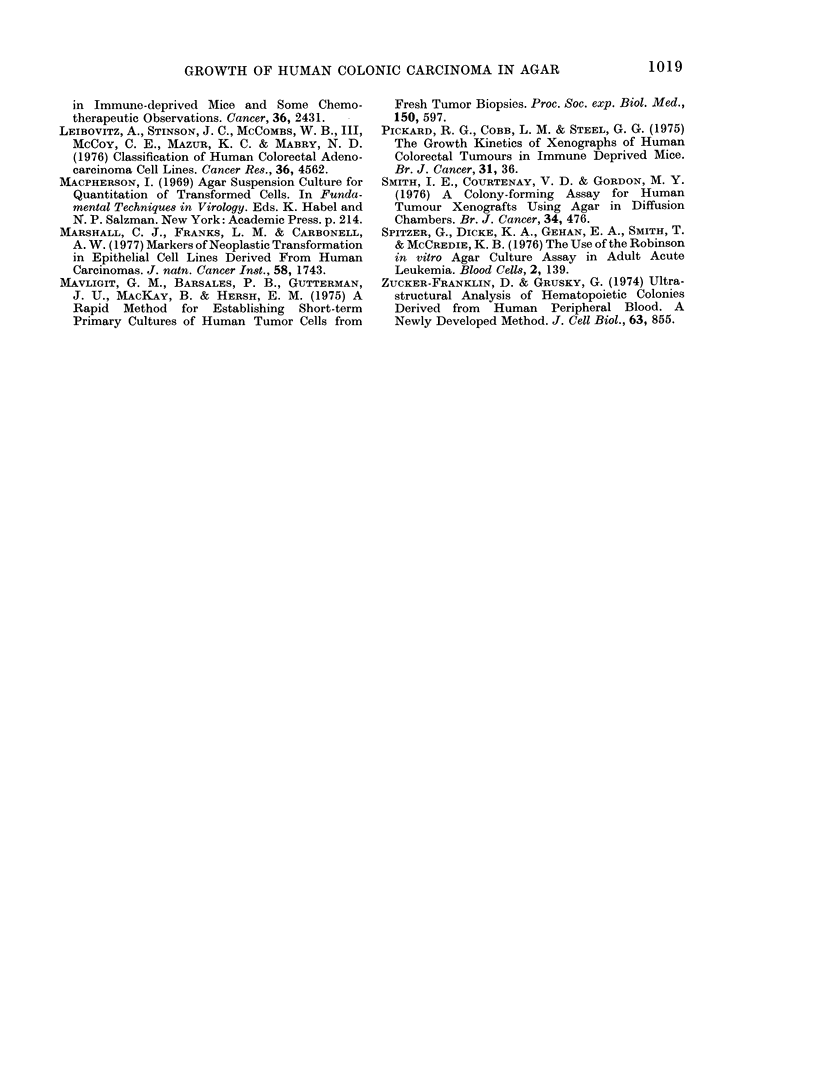# A soft-agar procedure measuring growth of human colonic carcinomas.

**DOI:** 10.1038/bjc.1978.147

**Published:** 1978-06

**Authors:** P. M. Kimball, M. G. Brattain, A. M. Pitts

## Abstract

**Images:**


Br. J. Cancer (1978) 37, 1015

### A SOFT-AGAR PROCEDURE MEASURING GROWTH OF HUMAN COLONIC CARCINOMAS

P. M. KIMBALL, M. G. BRATTAIN AND A. M. PITTS

*From the Departments of Pathology and Biochemistry, University of Alabama Medical Center, Birmingham, Alabama 35294, U.S.A.*

Received 9 December 1977 Accepted 8 February 1978

**Summary.—Cell suspensions from 5 human colonic carcinomas were fractionated by velocity sedimentation and plated in soft agar. Cluster formation was restricted to the purest fraction of epithelial cells, as had been determined by immuno- and histochemical criteria. Plating efficiencies for the 5 specimens were 1·0–4·5%. The effects of varying the incubation period and inoculum size upon growth were studied using unseparated cell suspensions from 6 specimens. Clusters were apparent after 3 weeks in culture, and maximum cluster formation was typically seen by 5 weeks. Cluster formation appeared concentration-dependent, and individual specimens varied with respect to the inoculum most conducive to growth. The maximum plating efficiencies for unseparated cells were 0·4–1·7%.**

THE STUDY of cytotoxic agents on human colonic carcinoma would benefit by the development of a system which (1) quantitated tumour cell growth and (2) was applicable to most specimens. The difficulty of establishing cell cultures from primary colonic carcinomas is well known (Leibovitz *et al.*, 1976). Additionally, those specimens established as cell lines may alter with passage, and the response to drug therapy may not reflect the original tumour (Smith, Courtenay and Gordon, 1976; Lamerton and Steel, 1975). The establishment and maintenance of human tumour xenographs presents similar problems (Pickard, Cobb and Steel, 1975; Smith *et al.*, 1976; Lamerton and Steel, 1975).

Growth in agar has been described as a trait of malignant transformation (Macpherson, 1969; Marshall, Franks and Carbonell, 1977). Recently, Smith *et al.* (1976) reported a quantitative assay of growth in agar, using cells from human tumour xenographs. We wish to report the development of a soft-agar procedure for measuring growth of primary human colonic carcinomas. The effects of inoculum size and varied periods of incubation upon cluster formation were studied.

### METHODS

*Cell suspensions.*—Colonic carcinomas were digested with 0·25% trypsin (Microbiological Associates, Bethesda, Md.) and the resultant cell suspensions were stored over liquid $N_2$ as previously reported (Kimball *et al.*, 1976; Brattain *et al.*, 1977*a*,*b*,*c*). Before culture, the frozen cells were rapidly thawed at 37°C and diluted with McCoy's enriched medium with 20% foetal calf serum and antibiotics (4·3 $\mu$g/ml gentamicin, 90 $\mu$g/ml streptomycin, 90 $\mu$g/ml penicillin). Cells were sedimented at 97 *g* for 7·5 min, resuspended in McCoy's medium, and forced through Nitex (TETKO, Inc., Elmsford, N.Y.,) with a pore diameter of 48 $\mu$m. Cell counts were performed with haemocytometer chambers, and viability was assessed by trypan-blue exclusion. The percentage of nucleated cells was determined from Wright's stains of cytocentrifuge preparations of the cell suspensions.

Reprint requests to Dr Michael G. Brattain, Department of Biochemistry, University of Alabama Medical Center, Box 16, University Station, Birmingham, Alabama 35294.

Initial experiments were conducted with cells purified by velocity sedimentation as previously described (Brattain *et al.*, 1977*b*,*c*). Briefly, cells were sedimented in a linear density gradient of Ficoll (Pharmacia Fine Chemicals, Inc., Piscataway, N.J.) in tissue-culture medium and collected in 4 fractions. Fractions I–III (consisting of the upper 75% of the gradient) contained primarily red blood cells, granulocytic and agranulocytic leucocytes. Compared to the starting sample suspension, Fraction IV (comprising the lower 25% of the gradient) contained a 2- to 3-fold increase in the concentration of epithelial cells, as demonstrated by the histochemical marker, N-acetylglucosaminidase, and increased amounts of carcino-embryonic antigen. The morphology of the cells in the various fractions have been described (Brattain *et al.*, 1977*c*). Each fraction was sedimented at 97 *g* for 7·5 min. The cell pellet was resuspended in McCoy's medium and prepared for culture as described above.

*Agar culture.*—Cells were suspended in a final concentration of 0·27% agar (Matheson, Coleman and Bell, Norwood, Ohio) containing antibiotics. One ml of this suspension was layered over a base layer of 0·5% agar containing antibiotics in 35mm plastic Petri dishes (Falcon 3001, Fisher Scientific Co., Norcross, Ga.) (Macpherson, 1969). Agar cultures were incubated at 37°C under humidified conditions and 5% $CO_2$ in air. Duplicate cultures were terminated at specific intervals and embedded in plastic (Zucker-Franklin and Grusky, 1974). Cell growth was scored microscopically. We have observed during purification and culturing procedures that cells from primary human colonic carcinomas rapidly form small aggregates which are not effectively removed by filtration through fine-mesh Nitex. Appropriate controls were counted to determine the number of aggregates initially present in the culture. Cluster formation is expressed as the percent plating efficiency (PE):

$$\frac{(\text{number of clusters} - \text{initial aggregates}) \times 100}{\text{number of viable nucleated cells plated}}.$$

### RESULTS

Cell suspensions from 5 human colonic carcinomas were separated by velocity sedimentation, and the resultant fractions plated in soft agar as described in the Methods section. After 4–5 weeks' incubation, the formation of clusters consisting of 7 or more cells was restricted to Fraction IV (Table I). Because clusters of this size were confined to the purest fractions of epithelial cells, a cluster was subsequently defined as consisting of a minimum of 7 cells, unless otherwise specified in the text. Cluster formation was apparent in Fraction IV of all specimens (Brattain *et al.*, 1977*b*,*c*. Inocula for the 5 specimens ranged from 10 to 50,000 cells and the percent cluster formation varied from 1·0 to 4·5%. Comparison of the data suggested that the relationship between inoculum and resulting PE differed between specimens.

Unseparated cell suspensions from 6 specimens were used to determine the effects of cell concentration and incubation period upon cluster formation. Cluster formation was observed in all specimens after 3 weeks' incubation and maximum cluster formation was usually apparent by 5 weeks (Table IIA). A breakdown of cluster structure was frequently seen by the 7th week in culture. The maximum PE with unseparated cells seldom exceeded 1·0% (Table 11A). There was considerable variation in the amount of cluster formation between specimens. Each specimen demonstrated concentration-dependent growth; however, the inoculum most conducive to cluster formation varied between tumours.

The purified Fraction IV from 2 specimens was studied as described for unseparated cells (Table IIB). Both specimens demonstrated that cluster formation was greatest after 5 weeks in culture and had declined by the 7th week. Concentration studies were possible with only one specimen which showed concentration-dependent cluster formation.

Generally, two types of clusters were observed in the cultures of separated and unseparated cell suspensions. All specimens contained clusters in which the cells formed tightly bound spheroid structures (Fig.). Several specimens additionally contained clusters with a loose and less

TABLE I.—*Soft-agar Growth of the Cell Fractions Obtained from Velocity Sedimentation*

| Fraction | % PE 2 Cells/cluster | 3–6 Cells/cluster | ≥ 7 Cells/cluster |
|---|---|---|---|
| I | 1·1 | 0·1 | 0 |
| II | 0 | 0 | 0 |
| III | 2·2 | 1·3 | 0 |
| IV | 8·6 | 7·3 | 1·4 |

Cells from a human colonic carcinoma were separated by velocity sedimentation and collected in 4 fractions. Each fraction was plated in soft agar with an inoculum of 50,000 viable nucleated cells. Cultures were terminated at 4 weeks and embedded in Epon plastic (Zucker-Franklin and Grusky, 1974). The formation of cell clusters was scored microscopically. Growth is expressed as the % PE of the variously sized clusters.

spheroid configuration. When present, the loosely bound clusters occurred in all inocula and were seen at the earliest termination of the cultures which suggested that they were not a product of spheroid cluster degradation. Cluster formation was observed in all specimens. Several specimens additionally formed large colonies. We have not yet determined whether the variations in cluster morphology and growth will be of use in the prediction of therapeutic response, as has been suggested with leukaemic progenitor cells (Spitzer *et al.*, 1976).

### DISCUSSION

Many malignant cells have demonstrated the ability to grow in semi-solid media (Macpherson, 1969; Marshall *et al.*, 1977). While recent reports have suggested the possible use of agar culture in studying and predicting the therapeutic response of neoplastic cells, relatively little work has been performed with primary tumour specimens (Spitzer *et al.*, 1976; Smith *et al.*, 1976). Smith *et al.* (1976) indicated that a reproducible agar assay would be difficult to develop for primary tumours, due to the lack of repeated biopsy samples from the same patient. The ability to store cells over liquid $N_2$ enabled us to develop a reproducible quantitation of the growth of primary human colonic carcinomas in agar. The criterion for cluster formation was established by preliminary work with cells purified by velocity sedimentation (Brattain *et al.*, 1977*b*,*c*). Cluster formation was observed in all specimens cultured in agar. The initial rate of cluster formation was consistent with that described for colonic lines and colon tumour xenographs cultured in agar (Smith *et al.*, 1976; Marshall *et al.*, 1977). The decrease in cluster frequency observed

FIG.—Typical agar clusters from a human colonic carcinoma after 5 weeks in culture. ×75.

TABLE II.—*Growth of Human Colonic Carcinoma Cells in Agar as a Function of Inoculum Size and Incubation Period*

| | Specimen | Inoculum (× $10^4$) | % Cluster formation 3 weeks | 5 weeks | 7 weeks |
|---|---|---|---|---|---|
| (A) | A | 1 | 0·10 | 0·56 | 0 |
| | | 4 | 0·08 | 0·13 | 0 |
| | | 8 | 0·06 | 0·16 | 0·08 |
| | B | 1 | 0 | 0 | 0 |
| | | 4 | 0 | 0 | 0 |
| | | 8 | 0·07 | 0·90 | 0·90 |
| | C | 1 | 0·20 | 1·00 | N.D.* |
| | | 4 | 0·20 | 0·60 | N.D. |
| | | 8 | 0·05 | 0·01 | N.D. |
| | D | 1 | 1·70 | 0·80 | 0 |
| | | 4 | 0·50 | 0·60 | 0·50 |
| | | 8 | 0·80 | 0·80 | 0·50 |
| | | 16 | 0 | 0·40 | 0·30 |
| | E | 1 | 0·10 | 0·20 | 0·30 |
| | | 4 | 0·03 | 0·30 | 0·60 |
| | | 8 | 0·20 | 0·20 | 0·05 |
| | | 16 | 0·02 | 0·01 | 0·20 |
| | F | 1 | N.D. | 0·05 | 0 |
| | | 4 | 0·40 | 0 | 0 |
| | | 8 | 0·30 | 0·30 | 0 |
| | | 16 | N.D. | 0·40 | 0·15 |
| (B) | E | 4 | 0·70 | 1·80 | 1·30 |
| | | 10 | 0 | 0·40 | N.D. |
| | G | 1 | 0·10 | 1·00 | 0·50 |

(A) Cells from 6 human colon tumours were plated in soft agar at the indicated inocula of viable nucleated cells. Viability was 68–88%. Cultures of each inoculum were terminated at 3, 5 and 7 weeks and embedded in plastic (Zucker-Franklin and Grusky, 1974). Growth is expressed as the percent cluster formation (a cluster consists of $\geq$ 7 cells). Duplicate cultures varied by ~15%.

(B) After velocity sedimentation Fraction IV from 2 specimens was plated in soft agar. Cultures were terminated and counted as described for A.

* N.D. = not determined.

with several specimens after 7 weeks in culture suggested cluster death, which may have been caused by depletion of essential nutrients, accumulation of metabolic products, alterations in agar consistency or the attainment of a terminal stage by the proliferating cells. The changes in cluster frequency with time occurred in cultures of both separated and unseparated cell suspensions.

Several specimens were plated in agar before and after velocity centrifugation. Based on the degree of purification, the PE of the purified cell suspension was consistently greater than was predicted by the growth of an equivalent inoculum of unseparated cells from the same specimen (Brattain *et al.*, 1977*b*, *c*). Mavligit *et al.* (1975) have suggested that a large percentage of non-viable cells may inhibit *in vitro* growth. Although initial proportions of viable cells were equivalent, the unseparated cell suspension may have suffered a higher incidence of cell death during the lengthy incubation period. The low PEs observed with many specimens might be improved by studies to determine optimal growth conditions.

All specimens showed concentration-dependent growth. Specimens varied with respect to PE and the inoculum optimal for growth. This may reflect differences in the proportion or nature of malignant cells or of those populations capable of proliferation in agar. The similar results obtained from duplicate cultures does not eliminate the possibility of some random variation. We have not yet determined which of the various cell types observed in suspensions of colonic carcinoma are responsible for growth in agar (Brattain, *et al.* 1977*c*). Studies are in progress to determine whether this procedure will form the basis of a useful assay in the study of cytotoxic agents on human colonic carcinoma.

The authors wish gratefully to acknowledge the support of Dr T. G. Pretlow II during the course of this work.

Supported by Public Health Service Grants CA-15089, CA-16764, and CA-16430 from the National Cancer Institute, DE-2670 from the National Institute of Dental Research, and by American Cancer Society Grant PDT-9B.

### REFERENCES

BRATTAIN, M. G., KIMBALL, P. M. & PRETLOW, T. G., II. (1977*a*) β-Hexosaminidase Isozymes in Human Colonic Carcinoma. *Cancer Res.*, **37**, 731.

BRATTAIN, M. G., KIMBALL, P. M., PRETLOW, T. G., II & PITTS, A. M. (1977*b*) Partial Purification of Human Colonic Carcinoma Cells by Sedimentation. *Br. J. Cancer*, **35**, 850.

BRATTAIN, M. G., PRETLOW, T. G. & PRETLOW, T. G., II. (1977*c*) Cell Fractionation of Large Bowel Cancer. *Cancer*, **40**, 2479.

KIMBALL, P. M., BRATTAIN, M. G., PRETLOW, T. G., II & PITTS, A. M. (1976) The Purification of Human Colonic Tumor Cells. *Fed. Proc.*, **35**, 758.

LAMERTON, L. F. & STEEL, G. G. (1975) Growth Kinetics of Human Large Bowel Cancer Growing in Immune-deprived Mice and Some Chemotherapeutic Observations. *Cancer*, **36**, 2431.

LEIBOVITZ, A., STINSON, J. C., MCCOMBS, W. B., III, MCCOY, C. E., MAZUR, K. C. & MABRY, N. D. (1976) Classification of Human Colorectal Adenocarcinoma Cell Lines. *Cancer Res.*, **36**, 4562.

MACPHERSON, I. (1969) Agar Suspension Culture for Quantitation of Transformed Cells. In *Fundamental Techniques in Virology*. Eds. K. Habel and N. P. Salzman. New York: Academic Press. p. 214.

MARSHALL, C. J., FRANKS, L. M. & CARBONELL, A. W. (1977) Markers of Neoplastic Transformation in Epithelial Cell Lines Derived From Human Carcinomas. *J. natn. Cancer Inst.*, **58**, 1743.

MAVLIGIT, G. M., BARSALES, P. B., GUTTERMAN, J. U., MACKAY, B. & HERSH, E. M. (1975) A Rapid Method for Establishing Short-term Primary Cultures of Human Tumor Cells from Fresh Tumor Biopsies. *Proc. Soc. exp. Biol. Med.*, **150**, 597.

PICKARD, R. G., COBB, L. M. & STEEL, G. G. (1975) The Growth Kinetics of Xenographs of Human Colorectal Tumours in Immune Deprived Mice. *Br. J. Cancer*, **31**, 36.

SMITH, I. E., COURTENAY, V. D. & GORDON, M. Y. (1976) A Colony-forming Assay for Human Tumour Xenografts Using Agar in Diffusion Chambers. *Br. J. Cancer*, **34**, 476.

SPITZER, G., DICKE, K. A., GEHAN, E. A., SMITH, T. & MCCREDIE, K. B. (1976) The Use of the Robinson *in vitro* Agar Culture Assay in Adult Acute Leukemia. *Blood Cells*, **2**, 139.

ZUCKER-FRANKLIN, D. & GRUSKY, G. (1974) Ultrastructural Analysis of Hematopoietic Colonies Derived from Human Peripheral Blood. A Newly Developed Method. *J. Cell Biol.*, **63**, 855.